# Targeting Bacterial Sortases in Search of Anti-virulence Therapies with Low Risk of Resistance Development

**DOI:** 10.3390/ph14050415

**Published:** 2021-04-30

**Authors:** Georgiana Nitulescu, Denisa Margina, Anca Zanfirescu, Octavian Tudorel Olaru, George Mihai Nitulescu

**Affiliations:** Faculty of Pharmacy, “Carol Davila” University of Medicine and Pharmacy, Traian Vuia 6, 020956 Bucharest, Romania; georgiana.nitulescu@umfcd.ro (G.N.); denisa.margina@umfcd.ro (D.M.); anca.zanfirescu@umfcd.ro (A.Z.); octavian.olaru@umfcd.ro (O.T.O.)

**Keywords:** Gram-positive pathogens, *Staphylococcus aureus*, *Streptococcus mutans*, covalent inhibitors, sortase A inhibitors, flavonoids, cinnamic acid derivatives, quinones, thiadiazoles, triazolothiadiazoles

## Abstract

Increasingly ineffective antibiotics and rapid spread of multi- and pan-resistant bacteria represent a global health threat; hence, the need of developing new antimicrobial medicines. A first step in this direction is identifying new molecular targets, such as virulence factors. Sortase A represents a virulence factor essential for the pathogenesis of Gram-positive pathogens, some of which have a high risk for human health. We present here an exhaustive collection of sortases inhibitors grouped by relevant chemical features: vinyl sulfones, 3-aryl acrylic acids and derivatives, flavonoids, naphtoquinones, anthraquinones, indoles, pyrrolomycins, isoquinoline derivatives, aryl β-aminoethyl ketones, pyrazolethiones, pyridazinones, benzisothiazolinones, 2-phenyl-benzoxazole and 2-phenyl-benzofuran derivatives, thiadiazoles, triazolothiadiazoles, 2-(2-phenylhydrazinylidene)alkanoic acids, and 1,2,4-thiadiazolidine-3,5-dione. This review focuses on highlighting their structure–activity relationships, using the half maximal inhibitory concentration (IC_50_), when available, as an indicator of each compound effect on a specific sortase. The information herein is useful for acquiring knowledge on diverse natural and synthetic sortases inhibitors scaffolds and for understanding the way their structural variations impact IC_50_. It will hopefully be the inspiration for designing novel effective and safe sortase inhibitors in order to create new anti-infective compounds and to help overcoming the current worldwide antibiotic shortage.

## 1. Introduction

The worldwide increasing resistance of bacterial pathogens to antibiotics imposes alternative strategies to treat infectious diseases [1,2]. Drug resistant bacteria pose a great threat and more and more infections fail to respond to available treatments [3]. Methicillin-resistant *Staphylococcus aureus*, vancomycin-resistant *Enterococcus*, and carbapenem-resistant *Enterobacteriaceae* are just some examples of major resistant bacteria that cause serious diseases sometimes resulting in the death of the patients [4]. The identification and analysis of the bacterial mechanisms of pathogenicity emerged as a promising strategy for drug development. The virulence factors are used by pathogens to colonize, invade, and persist within a susceptible host, surviving its immune system [5]. Bacteria use virulence factors to disable the host immune system and to invade its tissues. Drugs blocking these factors without killing the bacteria create less evolutionary pressure and diminish the chances of resistant genes to emerge. Various strategies are studied for implementation into future therapies, like the inhibition of the quench pathogen quorum sensing (QS) systems, disrupting the biosynthesis of the functional membrane microdomains, the inhibition of the biofilm formation, and toxins’ neutralization [6].

The center of attention for this paper is one of the most promising anti-virulence strategies targeting prominent Gram-positive pathogens: the inhibition of bacterial sortases (Srt) using small molecules [5]. Even if the majority of Gram-positive bacteria contain at least one Srt enzyme, this work was focused on pathogens with a high risk for human health and with a low range of therapeutic solutions available, like *Staphylococcus aureus* (*S. aureus*), *Staphylococcus epidermidis* (*S. epidermidis*), *Streptococcus mutans* (*S. mutans*), *Streptococcus pneumoniae* (*S. pneumoniae*), *Streptococcus pyogenes* (*S. pyogenes*), *Bacillus anthracis* (*B. anthracis*), or *Listeria monocytogenes* (*L. monocytogenes*), highlighting therapeutically promising Srt inhibitors.

## 2. Sortases’ Role in Virulence Mechanisms

Gram-positive bacteria are surrounded by a cell wall containing a series of attached surface polypeptides and polysaccharides, like streptococcal M protein and staphylococcal protein A [7], which have multiple roles such as adhesion to host tissues, evasion of host defences, and biofilm formation [8]. A screening for *S. aureus* mutants that cannot cleave the protein A identified that bacteria defective in the anchoring of surface proteins is carrying a mutation in the srtA gene [9]. This gene encodes Srt, an enzyme of 206 aminoacids with an N-terminal membrane-spanning domain and a C-terminal catalytic domain. The lack of Srt is associated with the inability to bind the surface proteins that anchor at their C-terminal ends [10] and thus, the surface adhesion is abolished and the process of establishing an infection is reduced [11].

Having a key role in the attachment of surface proteins to the cell wall, these enzymes became important targets in the search for anti-virulence drugs. Early studies demonstrated that the cell wall sorting reaction depends on an active thiol group that is sensitive to agents that can interact with it [12]. Later, it was demonstrated that in *S. aureus*, the SrtA contains a cysteine (Cys) at position 184, which is critical for the enzyme activity [13].

There are more than 1800 gene sequences that encode Srt enzymes that have been identified in almost 600 species of bacteria. Most of them express more than one type of Srt that function to attach distinct proteins to the cell surface [14]. They are not essential for bacterial viability, emerging therefore as anti-virulence drug targets [15].

The sortases characterized so far are part of the cysteine transpeptidase class and catalyse the reaction that binds a peptide with a sorting signal consisting of a LPXTG motif to the nucleophilic amino group within the cell wall sorting signal (CWSS) of the protein. Due to the variation of their sorting signal, the specificities of the nucleophilic substrate, and also their role, the Srt family includes six classes (A–F) [15].

The most studied are the class A Srt with their representative *S. aureus* SrtA (Sa-SrtA). They are known as “housekeeping enzymes” because they anchor a large number of functionally distinct proteins to the cell wall by recognizing the sorting signals that contain the LPXTG consensus [16]. The class B of Srt (SrtB) cleave surface protein at an NPQTN signal and are involved in iron metabolism [17].

## 3. Screening Protocols for Identification of Sortases Inhibitors

There are several protocols for the assessment of the inhibitors’ effect on sortases. The most used methods rely on measuring the fluorescence intensity upon the enzymatic cleavage of a synthetic peptide substrate.

The wild type staphylococcal SrtA has a low solubility rendering its use difficult. A truncated soluble form, named SrtAΔ24 and presented here as Sa-SrtAΔ24, was obtained by removing the NH_2_-terminal membrane anchor segment (residues 2–25) using the *S. aureus* OS2’s chromosome expressed in *Escherichia coli* (*E. coli*) XL-1 Blue cells [12,13]. This truncated sortase was also obtained using PCR to amplify the genomic DNA of *S. aureus* ATCC 6538p in *E. coli* TOP10 cells [18]. Wand et al. (2015) developed Sa-SrtAΔN59, another truncated sortase that contains only residues 60–206 using genomic DNA from *S. aureus* Newman D2C [19]. In a similar manner, a truncated *S. aureus* sortase B, SrtBΔN30, was PCR-amplified by using primers that removed the NH_2_-terminal 30 amino acid residues representing the membrane anchor sequence [18].

In the case of *S. mutans*, used frequently is the NH_2_-terminal 40 amino acid truncated enzyme SrtAΔ40 derived from the strains OMZ65 [20] or UA159 [21]. *S. pneumoniae* D39 genomic DNA was used to obtain the truncated SrtA∆N81 (Spn-SrtA) [22]. The assays targeting *L. monocytogenes* usually employ the truncated SrtAΔN70 amplified using the DNA sequence encoding SrtA residues Ala71 to Lys221 and specific primers [23].

Gosschalk et al. (2020) developed and implemented a cell-based method to identify sortase inhibitors using *Actinomyces oris* (*A. oris*), based on its sortase-dependent culture growth. The advantage of this method is that it provides a realistic context for the intact enzyme inhibition in the natural microbial membrane [24]. However, the differences in *A. oris* sortase and Sa-SrtA could provide false positive hits.

The most important differences between the assay protocols, used for finding Srt inhibitors, can be observed in the nature of the used substrate. The method developed by Kim et al. (2003) uses Dabcyl-QALPETGEE-Edans (0.75 μg) as fluorogenic peptide substrate. The enzyme and the substrate are diluted in a mixture of 50 mM Tris-HCl buffer (pH 7.5), 150 mM NaCl, and 5 mM CaCl_2_ and the fluorescence is recorded at 495 nm after excitation at 350 nm [25]. A similar method was developed by Kruger et al. (2004) using as substrate 2-amino-benzoyl-LPETG-diaminopropionic acid-dinitrophenyl-NH_2_, also known as Abz-LPETGDap(Dnp)-NH_2_, and measuring the increase in fluorescence at 420 nm after excitation at 317 nm. The Abz-LPETGDap(Dnp)-NH_2_ substrate together with pentaglycine were used to develop a high-performance liquid chromatography (HPLC) assay in which the areas of peaks corresponding to the substrate and the reaction product (H-G-Dap(Dnp)-NH_2_) were measured by UV detection at 355 nm [26]. A commercially available kit contains a substrate that releases upon the enzymatic reaction 5-carboxyfluorescein (5-FAM) that can be monitored at emission 520 nm after excitation at 490 nm [27,28].

## 4. Sortase A Inhibitors

There are several works that present known Srt inhibitors, but most of them chose to classify these substances as natural and synthesis compounds [29,30]. We presented here an exhaustive collection of SrtA inhibitors grouped by relevant chemical features highlighting their structure–activity relationships, rather than using their source as classification criteria. The half maximal inhibitory concentration (IC_50_) presented here should be considered as a relative, and not an absolute, indicator of each compound’s effect on a specific Srt target. The differences in testing protocols can be important and should not be ignored. When available, the minimum inhibitory concentration (MIC) values are presented and represent the lowest tested concentration of the compound at which no bacteria growth was observed. Low Srt’s IC_50_ values coupled with high MIC values are an indicator of the compound’s potential to disrupt the pathogenesis of the bacteria without affecting its viability, placing no pressure on bacteria to develop drug-resistant mechanisms [31].

### 4.1. Plant Extracts

A screening of medicinal plant extracts as Sa-SrtA inhibitors reported the evaluation of 80 dried extracts and their corresponding *n*-hexane, ethyl acetate, and water fractions. The measured IC_50_ values were in the range of 1.5–39.4 μg/mL The best inhibitory effects were registered for the ethyl acetate fractions of *Cocculus orbiculatus* (syn. *Cocculus trilobus*, Fam. Menispermaceae) rhizome, *Liriope muscari* (syn. *Liriope platyphylla*, Fam. Asparagaceae) tuber, *Fritillaria verticillata* (Fam. Liliaceae) tuber, and *Toxicodendron vernicifluum* (syn. *Rhus verniciflua*, Fam. Anacardiaceae) bark [32]. The evaluation of the anti-virulence potential of these extracts is limited, because this study offers no information on extracts’ impact on the bacterial growth.

A methanol extract obtained from *Curcuma longa* (turmeric, Fam. Zingiberaceae) dried rhizomes (80 μg/mL) produced a 80% inhibition of SrtA *S. aureus* ATCC 6538p activity after 1 h of incubation at 37 °C [33]. An extract from the dried fruits of *Psoralea corylifolia* (Fam. Fabaceae) produced a 44.2% inhibition of the *S. mutans* SrtA (Sm-SrtA) at 100 μg/mL [34]. A series of compounds were isolated from these extracts and are described in this study based on their structures.

### 4.2. Thiol Reactive Reagents

**Figure 1:**
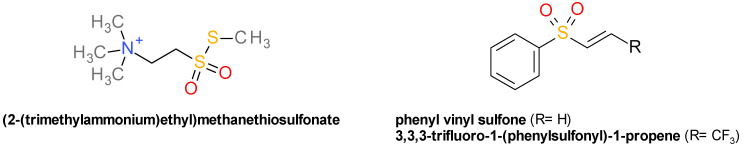
The structures of representative thiol reactive reagents and vinyl sulfones derivatives.

This group contains several chemical reagents know to interact with biologically active thiol groups that were identified as sortases inhibitors in the process of discovering the catalytic mechanism of the enzyme, but their broad and unselective reactivity profile renders them unfit for therapeutic development [35]. Methanethiosulfonates emerged as the first class of sortase inhibitors. Both (2-(trimethylammonium)ethyl)methanethiosulfonate (MTSET) and sodium (2-sulfonatoethyl)methanethiosulfonate (MTSES) interfere with the cleavage of sorting signals at the LPXTG motif [12] by forming a disulfide bond with the cysteine residue (Figure 1) [13]. Several cysteine proteases inhibitors were tested, identifying sodium *p*-hydroxymercuribenzoate (pHMB) as a sortase inhibitor, whereas alkylating reagents such as N-ethylmaleimide, iodoacetate, and iodoacetamide were not [12]. Sulfhydryl reducing agents, like dithiothreitol (DTT), had no effect on Sa-SrtA activity, but they reversed the inhibitory effect of MTSET by regenerating the thiol group [13].

### 4.3. Vinyl Sulfones

Several vinyl sulfones derivatives were evaluated against SrtAΔ24, based on their electrophilic potential to block active cysteine residues by forming thioether adducts through 1,4-addition reaction. The compound 3,3,3-trifluoro-1-(phenylsulfonyl)-1-propene exhibited the highest inhibitory effect, with an IC_50_ value of 190 µM and approximatively 1.5 folds higher MIC value (317.5 µM). The related phenyl vinyl sulfone has a significantly reduced inhibitory potency, with an IC_50_ of 736 µM, probably because of the lower electrophilic character (Figure 1). Phenyl trans-styryl sulfone had no inhibitory effect, indicating the importance of the molecule size. Mass spectrometry analysis confirmed the formation of a covalent bond between the phenyl vinyl sulfone and Sa-SrtA’s Cys184. Phenyl vinyl sulfone at 3000 µM reduced significantly the virulence of *S. aureus* Newman strain in a fibronectin binding model, without effecting the bacterial viability (MIC value over 6000 µM) [36].

The vinyl sulfone group was added to a scaffold of various *cis*-5-phenyl proline methyl esters, but the compounds exhibited only a modest activity towards SrtAΔ24, with IC_50_ values ranging from 850 µM to over 5000 µM [37].

### 4.4. 3-Aryl Acrylic Acids and Derivatives

Our analysis of the known sortases inhibitors revealed the 3-aryl substituted acrylic acid as a common and important scaffold, and therefore, we chose it as criteria for clustering them together in this group. The phenylacrylic acid, better known as cinnamic acid, is the structural core of various natural aromatic carboxylic acids with important roles in the biosynthesis of phenyl-propanoids, coumarins, lignans, flavonoids, stilbenes, aurones, anthocyanins, spermidines, and tannins [38]. This group contains natural and synthetic cinnamic acid derivatives and related compounds, as well as bioisosteric analogues.

#### 4.4.1. Cinnamic Acid Derivatives

A screening for inhibitors of the SrtA derived from *S*. *mutans* strain OMZ65 on an extract from the dried flowers of *Styphnolobium japonicum* (syn. *Sophora japonica*, Japanese pagoda tree, Fam. Fabaceae) provided two derivatives of the *p*-coumaric acid (4-hydroxycinnamic acid), maltol 3-*O*-(4′-*O*-*trans*-*p*-coumaroyl-6′-*O*-(3-hydroxy-3-methylglutaroyl))-*β*-glucopyranoside and its isomer, maltol 3-*O*-(4′-*O*-*cis*-*p*-coumaroyl-6′-*O*-(3-hydroxy-3-methylglutaroyl))-*β*-glucopyranoside. The derivative of *trans*-*p*-coumaric acid (conformer *Z*) exhibited an IC_50_ value of 58.6 μM, approximately 1.6 times more potent than the corresponding *cis*-*p*-coumaric acid derivative (conformer *E*). Both compounds exhibited no growth inhibitory activity on *S. mutans* OMZ65, the MIC values being higher than 345 μM [39].

A bioactivity-guided fractionation and separation of a *Pulsatilla koreana* (Korean pasque flower, Fam. Ranunculaceae) extract yielded several lignans and cinnamic acids. The highest inhibitory effects on the SrtAΔ40 derived from *S. mutans* strain OMZ65 were registered for 3,4-dihydroxycinnamic acid, also known as caffeic acid (IC_50_ = 20.2 μM) and its two ester derivatives, (−)-rosmarinic acid (IC_50_ = 20.1 μM) and (+)-chicoric acid (IC_50_ = 60.1 μM). The 3-*O*-methylated derivate of caffeic acid, the ferulic acid produced a low inhibitory effect on SrtA, with an IC_50_ over 100 μM [40]. Its isomer, 4-*O*-methylated derivate, the isoferulic acid was tested against SrtAΔN59 with a low effect, the measured IC_50_ being over 500 μM [41].

Another ester derivative of caffeic acid, the chlorogenic acid (3-*O*-caffeoylquinic acid), was identified as a potent inhibitor against SrtA from *S. aureus* Newman D2C. It exhibited an IC_50_ of 95.57 μM, without inhibition of bacterial cell growth (MIC greater than 1024 μg/mL). In mice studies, chlorogenic acid significantly interfered in the pathogenesis of *S. aureus* and prevented renal abscess formation [19]. Two isomers of the chlorogenic acid, cryptochlorogenic acid (4-*O*-caffeoylquinic acid) and neochlorogenic acid (5-*O*-caffeoylquinic acid), were tested against SrtAΔN59. Both isomers exhibited an IC_50_ over 500 μM, highlighting the importance of the functional groups’ reciprocal positions [41].

Three major constituents of the *Curcuma longa* (turmeric, Fam. Zingiberaceae) rhizome were found to inhibit SrtAΔ24 from *S. aureus* ATCC 6538p. The highest inhibitory effect was produced by curcumin with an IC_50_ value of 37.5 μM, followed by demethoxycurcumin (IC_50_ = 70.3 μM) and bisdemethoxycurcumin (IC_50_ = 103.5 μM). All three compounds showed no significant growth inhibitory activity against *S. aureus* strain Newman, with MIC values over 200 μg/mL [33]. Curcumin was found to be active also against *S. mutans* UA159 SrtA, with an IC_50_ value of 10.2 μM, a value considerably lower than the registered MIC value of 125 μM [21]. Curcumin can be considered a dimer of ferulic acid, compound with low inhibitory effect of *S. mutans* SrtA, indicating the complexity of the structure–activity relationships in this chemical class. Due to the presence of the *β*-diketo moeity, a Michael acceptor group, curcumin is known to react with cysteine sulfhydryl groups [42] and is most likely its mechanism for SrtA inhibition.

**Figure 2:**
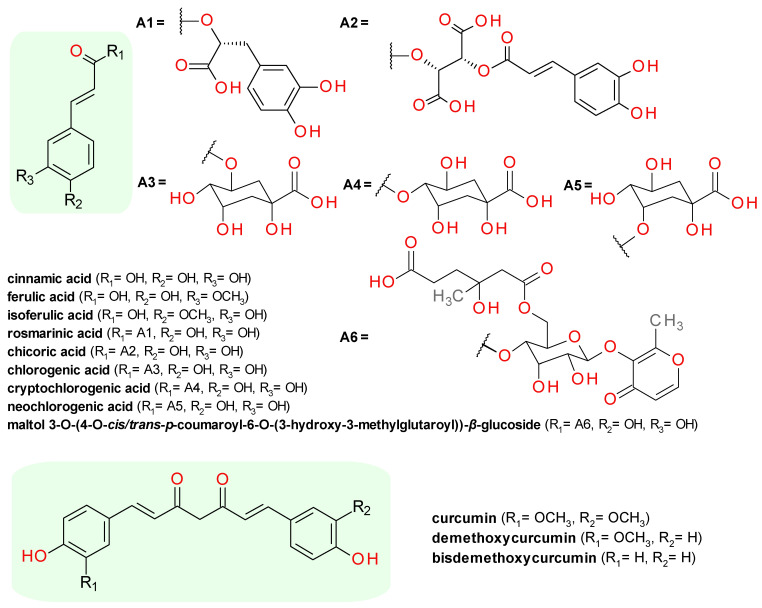
The structures of sortase inhibitors derivatives of cinnamic acid. In green, the general scaffold of each group.

The chemical structures of the cinnamic acid derivatives identified as Srt inhibitors are presented in Figure 2.

#### 4.4.2. Coumarin Derivatives

**Figure 3:**
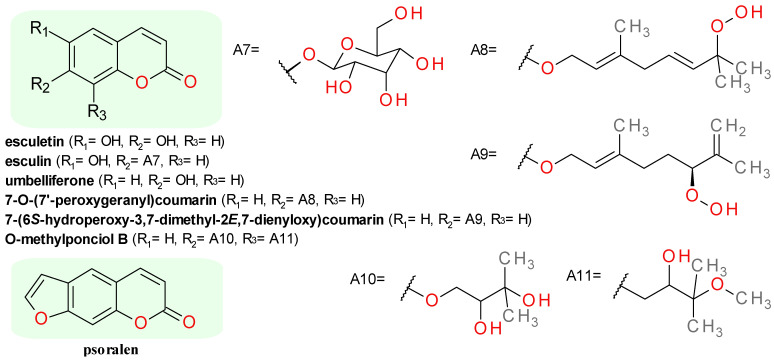
The structures of coumarin derivatives sortase inhibitors. In green, the general scaffold of each group.

Esculetin (6,7-dihydroxycoumarin, also known as aesculetin) is the lactone derivative formed by intramolecular cyclization of the 6-hydroxycaffeic acid. It inhibited Sa-SrtA with an IC_50_ value of 37.5 μM, while caffeic acid and its methoxy-derivative, the *trans*-ferulic acid, proved to be inactive. Esculetin exhibited no significant antimicrobial activity against *S. aureus* ATCC 25923 and *S. aureus* ATCC 6538. The impact of the 6,7-dihydroxy groups presence on the chromen-2-one scaffold was highlighted by the small inhibitory effect on Sa-SrtA produced by umbelliferone (7-hydroxycoumarin) and coumarin [28]. Psoralen, a furanocoumarin derivative of umbelliferone, had a low inhibitory effect on Sm-SrtA with IC_50_ close to 330 μM [34]. Esculin (esculetin 6-*O*-*β*-d-glucoside) caused low Sa-SrtA inhibition, probably because the 6-hydroxy group is blocked in an etheric bond [28]. The structures of the coumarin derivatives identified as Srt inhibitors are presented in Figure 3.

Several prenylated coumarins (Figure 3) were isolated from a dried fruits extract of *Poncirus trifoliate* (syn. *Citrus trifoliate*, trifoliate orange, Fam. Rutaceae) and tested as inhibitors on *S. aureus* ATCC6538p SrtA. The best effects were observed for 7-*O*-(7′-peroxygeranyl)coumarin (IC_50_ = 19.1 μM), 7-(6S-hydroperoxy-3,7-dimethyl-2E,7-dienyloxy)coumarin (IC_50_ = 23.5 μM), and O-methylponciol B (IC_50_ = 26.6 μM). The anti-infective potential of these compounds was demonstrated by an over 2-fold decrease of the fibrinogen cell clumping of *S. aureus* strain after 2 h of treatment with 100 μM. They also had no significant inhibitory effect on *S. aureus* ATCC6538p growth, the MIC values being over 300 μM [43].

#### 4.4.3. Aromatic Furanone Derivatives

**Figure 4:**
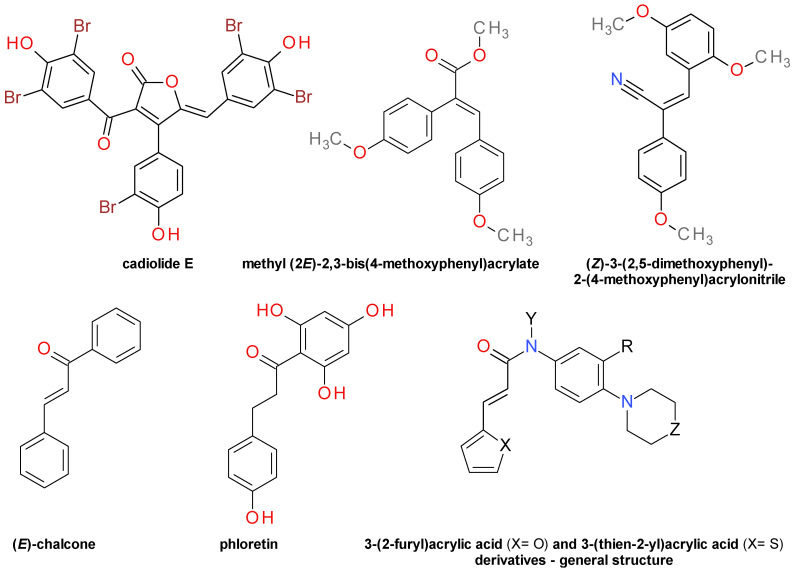
Aromatic furanone derivatives, diarylacrylonitriles, chalcones derivatives, 3-(2-furyl)acrylic acid, and 3-(thien-2-yl)acrylic acid derivatives.

A series of brominated tris-aromatic 2-furanones derivatives were isolated from the dark red ascidian *Synoicum sp.* (Fam. Polyclinidae) collected from the coast of southern Korea. Cadiolide E, a 4-(3-bromo-4-hydroxyphenyl)-2-furanone derivative (Figure 4), exhibited a significant inhibition on Sa-SrtA with an IC_50_ value of 78.8 µM, but also strong antibacterial effects. The related bis-aromatic diester, synoilide B, showed no significant effect on Sa-SrtA [44]. Structurally, both cadiolide E and synoilide B share a common fragment with the cinnamic acid.

A group of structurally related compounds named isocadiolides isolated from *Synoicum sp.* share the bromohydroxyphenyl fragments with the cadiolides, but differ in the nature of the central ring which is no longer a furanone, but one of the following structures: cyclopentene-1,3-dione, dihydrofuran, or pyranone. Isocadiolides A−D moderately inhibited Sa-SrtA, but also the bacterial development of *S. aureus* ATCC 6538P [45].

#### 4.4.4. Diarylacrylonitriles

A synthetic derivative of the *trans*-*p*-coumaric acid, the methyl (2*E*)-2,3-bis(4-methoxyphenyl)acrylate (Figure 4) was identified as hit in a random screening for a small-molecule Sa-SrtA inhibitors with an IC_50_ of 231 µM. The analysis of the structure–activity relationships revealed that the corresponding *Z*-conformer had close to 4-fold less inhibitory potency. The hydrolysis to the corresponding acid or the hydrogenation of the double bound both rendered the compound inactive. The bioisosteric replacement of the ester group with a nitrile significantly improved the Sa-SrtA inhibition. Contrary to the methyl acrylate derivatives, the potency of the diarylacrylonitrile derivatives was higher for the *Z*-isomers, the two benzene rings being in *trans* orientation [18].

Several structural modifications lead to (*Z*)-3-(2,5-dimethoxyphenyl)-2-(4-methoxyphenyl)acrylonitrile (DMMA) that reversibly inhibits Sa-SrtA with an IC_50_ of 9.2 µM [18]. DMMA (Figure 4) has been shown to inhibit also Sa-SrtB activity with an IC_50_ value of 34.2 µM and had no significant effect on *S. aureus* Newman strain’s growth, with MIC values over 678 µM [46]. The compound dose-dependently reduced the mortality rate in Balb/c mice infected with *S. aureus* Newman. Interestingly, the survival rate (75%) was lower for the mice treated intraperitoneally with 100 mg/kg/day, compared to those treated with 20 mg/kg/day (survival rate 100%) suggesting a toxic effect of DMMA itself. Nevertheless, DMMA proved to be effective (97% survival) even at 4 mg/kg/day [47].

#### 4.4.5. Chalcones Derivatives

Chalcones are derivatives of 1,3-diphenyl-2-propene-1-one that consist of two benzene rings linked by a three carbon unsaturated chain and can be considered cinnamoyl derivatives. (*E*)-chalcone is the basic member of the chalcone series [23]. The activity of (*E*)-chalcone against Sm-SrtA was measured and an IC_50_ of 5 μM was determined. The compound produced a slow and irreversible inhibition by forming a covalent adduct with the Cys205 residue through a Michael addition mechanism. This mechanism was demonstrated by the lack of inhibitory effects of the saturated analogue, dihydrochalcone [20]. Chalcone effectively inhibited Sa-SrtA (IC_50_ = 53.15 μM) without visibly altering *S. aureus* USA 300 growth (MIC > 4864 μM). The compound reduced the adhesion of *S. aureus* to fibronectin, biofilm formation, and bacterial invasion in a J774 mouse macrophages cells model. The *S. aureus* infected mice treated with subcutaneous injections of chalcone of 150 mg/kg at 12-h intervals had a higher 3-days survival rate compared to those untreated [48]. Isoliquiritigenin (2′,4,4′-trihydroxychalcone) isolated from an extract of the vine stem of *Spatholobus suberectus* (Fam. Fabaceae) inhibited Sa-SrtA with an IC_50_ of 139.7 μM [49].

(*E*)-Chalcone (Figure 4) inhibited also the *L. monocytogenes* SrtAΔN70 (Lm-SrtA) with an IC_50_ of 28.41 μM. The inhibition of SrtA activity reduced *L. monocytogenes* virulence and decreases the mortality of infected mice, with no visible influence on bacterial growth [23]. Interestingly, the dihydrochalcone derivative phloretin (Figure 4) effectively inhibited Lm-SrtA (IC_50_ = 37.24 μM) and produced no significant impact on *L. monocytogenes* growth (MIC = 933.44 μM). Molecular dynamics simulations indicated that the phloretin binds to Lm-SrtA through its hydroxyl groups in a region adjacent to the active enzymatic center [50]. This explains why the lack of the double bond is not important as in the case of the interaction of (*E*)-chalcone and Sm-SrtA.

#### 4.4.6. 3-(2-Furyl)Acrylic Acid and 3-(Thien-2-yl)Acrylic Acid Derivatives

The 3-(2-furyl)acrylic acid and its thiophene analogue can be considered bioisosters of the cinnamic acid. A series of phenyl-substituted amides derivatives of these acids (Figure 4) were synthesized and tested for their in vitro inhibitory effect against SrtAΔ59. The IC_50_ values ranged from 58 to 571 μM and for some compounds were described as over the 600 μM threshold. The hydrogenation of the acrylic double bond led to inactive compounds, thus underlining its importance. The N-methyl substituted amides proved to be weaker inhibitors than their corresponding NH analogues [51]. It is difficult to assess the anti-virulence potential of the compounds in this series in the absence of data regarding their impact on bacterial growth.

### 4.5. Flavonoids

**Figure 5:**
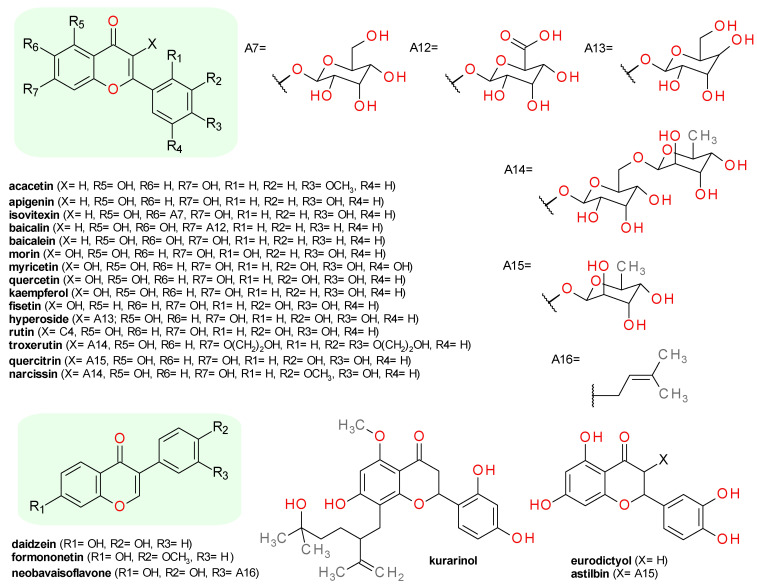
Structures of sortase inhibitors belonging to the flavonoids class. In green, the general scaffold of each group

Flavone (2-phenyl-4*H*-chromen-4-one) may be regarded as a closed ring derivative of (*E*)-chalcone. The flavonols are a subclass of flavonoids sharing a common 3-hydroxyflavone scaffold [52], where the 3-hydroxyl group seems to be essential for SrtA inhibition [28]. As the name suggests, the isoflavones are isomers of their corresponding flavones, differing in the position of the phenyl group on the chromen-4-one scaffold. Flavanones lack the double bond in the central ring and can be considered 2,3-dihydroflavones [52]. The chemical structures of Srt inhibitors belonging to this group are illustrated in Figure 5.

#### 4.5.1. Flavones

Acacetin (5,7-dihydroxy-4′-methoxyflavone) represents the 4′-*O*-methylated derivative of apigenin. It exhibited an IC_50_ of 128.3 μM on *S. aureus* SrtAΔN59 and a MIC value on *S. aureus* ATCC25904 greater than 3800 μM. A solution of 224 μM acacetin reduced almost to half the bacteria binding to fibrinogen, and in doses of 150 mg/kg/day, was able to protect mice from renal abscess induced by *S. aureus*, significantly increasing survival rates [53].

Isovitexin, the 6-C substituited glucoside of apigenin, inhibited Sa-SrtAΔN59 with an IC_50_ of 67.02 μM. The compound inhibited in a dose-dependent manner *S. aureus* cell adhesion to fibrinogen and biofilm formation without influencing the bacterial growth [54].

Baicalin, the 7-*O*-glucuronide of baicalein (5,6,7-trihydroxyflavone), inhibits Sa-SrtB with an IC_50_ value of 57.9 μM with no significant growth pressure on *S. aureus*. Baicalin reduced in a dose-dependent manner the adhesion of *S. aureus* to human alveolar epithelial A549 cells. The molecular dynamics simulations indicated the importance of both the chromen-4-one scaffold and the glucuronide moiety [55]. Baicalein inhibited in a concentration-dependent manner Lm-SrtA and attenuated *L. monocytogenes* entrance into Caco-2 cells cultures [56].

#### 4.5.2. Flavonols

A series of natural flavonols were isolated from an ethyl acetate extract of *Toxicodendron vernicifluum* bark and tested on SrtA and SrtB of *S. aureus* ATCC6538p using as substrate Dabcyl-QALPETGEE-Edans (for SrtA) and Dabcyl-NPQTN-Edans (for SrtB). In the case of SrtA, the best inhibition effects were registered for morin (3,5,7,2′,4′-pentahydroxyflavone, IC_50_ = 37.39 μM), myricetin (3,5,7,3′,4′,5′-hexahydroxyflavonol, IC_50_ = 44.03 μM), and quercetin (3,5,7,3′,4′-pentahydroxyflavone, IC_50_ = 52.70 μM). The measured IC_50_ values were generally lower on SrtB compared with those on SrtA, the most active compounds being morin (IC_50_ = 8.54 μM), kaempferol (3,5,7,4′-tetrahydroxyflavone, IC_50_ = 24.55 μM), and quercetin (IC_50_ = 33.28 μM). The MIC values on *S. aureus* Newman are over 300 μM for all these flavone derivatives [57]. The number and positions of the hydroxyl groups on the flavone scaffold seems to influence the inhibition of both SrtA and SrtB.

A number of flavonol derivatives were tested on SrtAΔ24 and a significant inhibitory effect were recorded only for myricetin with an IC_50_ value of 4.63 μM. Quercetin presented weak inhibitory effects at 10 μM, and its low water solubility hindered the testing at higher concentrations. Fisetin (3,7,3′,4′-tetrahydroxyflavone) had no significant effect [28]. The enzymatic assay coupled with molecular docking studies indicated that the flavonoid aglycones are more potent towards SrtA than their corresponding glycosides (hyperoside, rutin, and troxerutin), probably due to the lower number of rotatable bonds [28,58]. Quercetin significantly inhibited Spn-SrtA activity in a dose-dependent manner, but the IC_50_ values was not reported [22]. Quercitrin (syn. quercetin-3-rhamnoside), a quercetin glycoside derivative, inhibited the *S. aureus* SrtAΔN59 with an IC_50_ value of 72.77 μM, with little effect on Newman D2C strain growth [59].

The inhibitory effect of morin on SrtA from *S. mutans* was analyzed and the IC_50_ value was calculated as 27.2 µM. Furthermore, 30 µM morin significantly reduced biofilm formation of *S. mutans*, without changing the bacterial growth [60]. A bioactivity-guided separation of the *Styphnolobium japonicum* dried flowers extract yielded quercetin, rutin (quercetin 3-rutinoside), and narcissin (isorhamnetin 3-rutinoside). The compounds were tested against a recombinant SrtA derived from *S. mutans* strain OMZ65. Rutin exhibited an IC_50_ value of 134.1 μM, while its 3′-methylated derivate, narcissin presented an IC_50_ of 185.9 μM. The corresponding aglycone, quercetin, exhibited a weaker inhibitory effect, with an IC_50_ of 210.16 μM [61].

#### 4.5.3. Isoflavones

Several flavonoids isolated from *Spatholobus suberectus* (Fam. Fabaceae) were identified as potent inhibitors of Sm-SrtA without significant effects on *S. mutans* bacterial growth. The compounds were identified based on their spectroscopic data as two isoflavones, formononetin (7-hydroxy-4′-methoxyisoflavone, IC_50_ = 41.8 μM) and daidzein (4′,7-dihydroxyisoflavone, IC_50_ = 144.7 μM), and one flavone, 7-hydroxy-6-methoxyflavanone (IC_50_ = 46.1 μM) [62]. Neobavaisoflavone (3′-prenyldaidzein, IC_50_ = 284.1 μM) isolated from the dried fruits of *Psoralea corylifolia* presented a close to 2-folds lower inhibitory effect on Sm-SrtA compared to daidzein [34]. Formononetin also inhibited Sa-SrtA activity with an IC_50_ value of 74.9 μM, while its corresponding 7-*O*-*β*-d-glucoside had a 3-folds lower inhibitory effect [49].

#### 4.5.4. Flavanones

A series of seven prenylated flavonoids isolated from the roots of *Sophora flavescens* (shrubby sophora, Fam. Fabaceae) were evaluated for inhibitory activity against SrtA from *S. aureus* ATCC 6538p. All compounds produced a medium inhibitory effect, but the IC_50_ value could be calculated only for kurarinol, a derivative of 5-methoxyflavanone. Kurarinol has an IC_50_ value of 107 μM and a medium antibacterial activity against *S. aureus* (MIC = 219 μM) [63]. Eriodictyol (3,4,5,7-tetrahydroxyflavanone) inhibited Sa-SrtA with an IC_50_ value of 7.73 μM in a reversible mechanism and did not block *S. aureus* development even at a concentration 50-fold higher than IC_50._ The compound decreased in a dose-dependent manner the biofilm formation and the anchoring of the SpA protein in *S. aureus* [64].

#### 4.5.5. Flavanonols

Astilbin, the 3-*O*-rhamnoside of taxifolin (syn. dihydroquercetin) inhibits Sm-SrtA with an IC_50_ value of 16.6 μM without affecting the proliferation of *S. mutans* ATCC 25175. The molecular dynamics simulations reflected the role of the rhamnoside moiety in binding the enzyme active site. Astilbin produced a 49% reduction in bacterial biofilm formation at 142.1 μM, but no significant effect at 71 μM or lower concentrations. The authors consider that the biofilm inhibitory levels are much higher than IC_50_ for SrtA, because of the amount of not inhibited enzyme [65]. In our opinion, the compound’s low lipophilicity and cell permeability [66] is probably the cause of this difference.

### 4.6. Quinone Derivatives

**Figure 6:**
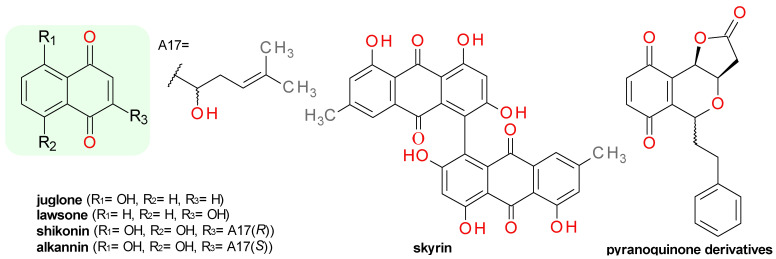
Sortase inhibitors derivatives of 1,4-quinone scaffold. In green, the general scaffold of each the naphtoquinones group.

A screening for SrtA inhibitors using a clinical drug and a natural product-based library and the monitoring of SrtAΔ24 cleavage of Abz-LPETG-Dnp substrate, identified two natural 5,8-dihydroxy-1,4-naphthoquinone derivatives, shikonin (IC_50_ = 0.30 μM) and alkannin (IC_50_ = 0.36 μM). The compounds are enantiomers and they both strongly inhibit the growth and viability of *S. aureus* [67] limiting considerably their use as anti-virulence agents. Similar 1,4-naphthoquinone derivatives, juglone (5-hydroxy-1,4-naphthalenedione) and its 2-methyl analogue, plumbagin, demonstrated a potent Sa-SrtA inhibitory effect with IC_50_ values of 1.78 µM and 16.71 µM respectively, without significantly influencing the bacterial growth of *S. aureus*, *E. faecalis*, and *S. epidermidis*. The inhibition kinetics and docking studies indicated an irreversible mechanism based on the Cys184’s thiol catalytic residue addition to the dual conjugated double system of the naphthoquinone structure. Lawsone, the 2-hydroxy-isomer of juglone, had no significant effect on Sa-SrtA at 10 μM because of its low reactivity towards nucleophilic agents [68]. The chemical structures of these naphthoquinone derivatives are presented in Figure 6.

Rhein, a natural anthraquinone derivative, chemically similar to juglone, demonstrated promising inhibitory effects at 10 µM, but its low water solubility hindered the assay for IC_50_ calculation [28]. Skyrin, a bisanthraquinone compound derived of two emodin (6-methyl-1,3,8-trihydroxyanthraquinone) moieties connected by a single C–C bond (Figure 6), exhibited Sa-SrtA inhibitory effects with IC_50_ values in the range of 24–31 µM. The anti-virulence potential is unfortunately reduced by the strong growth inhibitory activity against S. aureus Newman strain (MIC values around 10 µM) [69]. Emodin had little effect on Sa-SrtA at 100 µM [28].

A pair of pyranoquinone derivatives structurally related to the 1,4-naphthoquinones were identified to irreversibly inhibit Sa-SrtA with IC_50_ values of 6.2 μM and 19.4 μM by bonding to the nucleophilic thiol group of Cys184 in a Michael addition reaction. The two derivatives (Figure 6) are diastereomers differing in the stereochemistry of just one carbon atom, the isomer *R* being more active towards Sa-SrtA. Both compounds significantly attenuated the biofilm production at 80 μM and had no impact on the growth of *S. aureus* at 150 μM, demonstrating a good anti-virulence profile [67].

### 4.7. Indoles Derivatives

In the course of investigating an extract from tropical marine sponges from *Hyrtios* species (Fam. Thorectidae), a series of derivatives of 5-hydroxyindole were isolated [70] and tested for their effect on Sa-SrtA [71]. Serotonin (5-hydroxytryptamine) had no significant effect, but its N-closed ring analogue derivative, 1-carboxy-6-hydroxy-3,4-dihydro-*β*-carboline, demonstrated moderated inhibitory action against Sa-SrtA (IC_50_ = 290 µM). Based on its structure, a series of 3-indoleglyoxylic acid derivatives and their corresponding N-phenyl-amide were synthesized and evaluated as Sa-SrtA inhibitors. The compounds derived from indole and 2-methylindole, exhibited potent inhibitory properties with IC_50_ values in the range of 61 to 174 µM, while those derived from 5-hydroxy- and 5-methoxy-indole showed no significant effect. The related 3-glyceroindole and 3-indoleacetic acid were not active towards SrtA, indicating the importance of both carbonyl groups in the structure [71].

The screening of a series of indole derivatives and related heterocyclic compounds on *S. pyogenes* SrtA∆N81 (Sp-SrtA) led to the identification of (2-amino-6-chloro-1*H*-indol-3-yl)-morpholino-methanone with the corresponding IC_50_ value of 10 µM. The compound is structurally similar to the 3-indoleglyoxylic acid derivatives, but it had no significant effect on Sa-SrtA∆N59. The importance of the morpholino moiety for the inhibition of Sp-SrtA was demonstrated by docking studies and by the lack of effect of the corresponding piperidine derivative [72].

The Sa-SrtA inhibition assay-guided separation on a bright yellow sponge *Spongosorites* sp. (Fam. Halichondriidae) extract yielded several indole derivatives consisting of two indole moieties connected by a heterocyclic scaffold. Based on the nature of the joining heterocyle, the identified SrtA inhibitors can be classified as topsentins (bis(indolyl)imidazole derivatives) and hamacanthins (bis(indolyl)pyrazinone derivatives). From the first group, the best results were obtained for deoxytopsentin (IC_50_ = 48 µM), bromodeoxytopsentin (IC_50_ = 48 µM), bromotopsentin (IC_50_ = 39.7 µM), but they also had a major impact on *S. aureus* Newman bacterial growth. The hamacanthin derivatives had slightly less inhibitory potency than the related topsentins and strong antibacterial effects that limits their usefulness as anti-virulence potential therapies [73]. Two structurally related compounds, the spongosoritins B and C, sharing a central 2-methoxy-1-imidazole-5-one connecting ring, inhibited Sa-SrtA with IC_50_ values of 62.7 and 43.9 µM, respectively [74]. A series of bis(indolyl)-1,2,4-oxadizole derivatives were synthesized as topsentin analogues and their effects on *S. aureus* biofilm formation were evaluated. Three compounds were identified as potent biofilm inhibitors and all demonstrated a significant reduction in Sa-SrtA’s effect [75].

A number of six *β*-carboline alkaloids belonging to the eudistomin Y class were extracted and separated by multiple chromatographic methods from the marine ascidian *Synoicum sp.* (Fam. Polyclinidae) [76]. The *β*-carboline scaffold is based on an indole ring fused with a pyridine moiety [77]. The compounds were also isolated from a tunicate of the *Eudistoma* genus [78]. Only eudistomin Y4 (1-(3-bromo-4-hydroxybenzoyl)-6-bromo-β-carboline) and its isomer eudistomin Y5 presented inhibitory activity on Sa-SrtA with IC_50_ 163.2 μM and 146.4 μM, respectively. The compounds demonstrated also strong antibacterial effects on *S. aureus* ATCC 6538p with MIC values close to 7 μM and 14 μM, respectively. The hydrogenation of the carbonyl group to a hydroxyl group reduced considerably the Sa-SrtA inhibitory capacity [76].

A series of 1-phenyl-dihydro-*β*-carboline and 1-phenyl-tetrahydro-*β*-carboline derivatives were synthesized and some of them showed good inhibitory activity against SrtA, with IC_50_ values in the range of 25 to 115 μM. The majority of these compounds had no impact on *S. aureus* bacterial viability [71].

**Figure 7:**
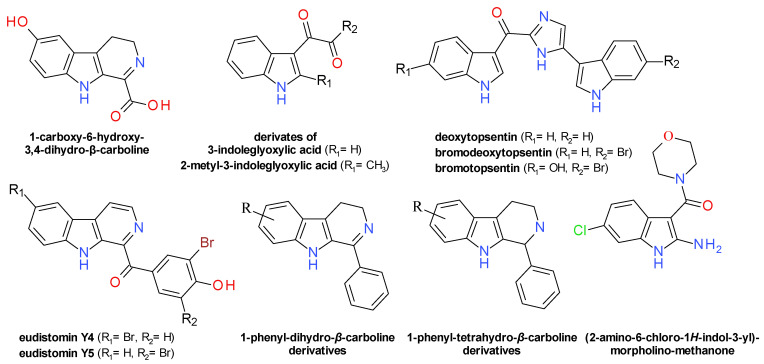
Sortase inhibitors derivatives of the indole scaffold.

The structures of the indole derivatives identified as Srt inhibitors are presented in Figure 7.

### 4.8. Pyrrolomycins and Analogues

**Figure 8:**
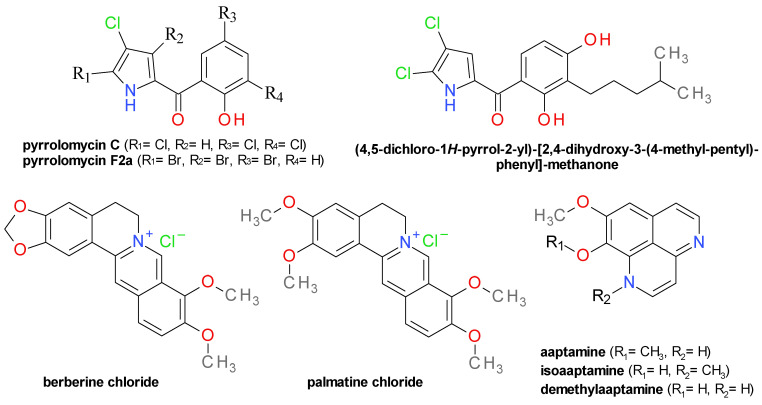
Pyrrolomycins and analogues. Isoquinoline derivatives.

Pyrrolomycins are halogenated antibiotics isolated from the fermentation broth of *Actinosporangium sp.* and *Streptomyces sp.* [79]. The natural pyrrolomycins C and F2a (Figure 8) moderately inhibited Sa-SrtA with IC_50_ values of 300 µM and 250 µM, respectively. Both compounds have potent antibacterial activities on *S. aureus* with MIC values under 1 µM [79]. Four synthetic derivatives were tested on Sa-SrtA and showed IC_50_ values in the range of 130–160 µM and strongly disrupted staphylococcal biofilm formation [79]. The structurally related compound, (4,5-dichloro-1*H*-pyrrol-2-yl)-[2,4-dihydroxy-3-(4-methyl-pentyl)-phenyl]-methanone (Figure 8), produced Sa-SrtA (IC_50_ = 47 µM) inhibition along with a reduction in biofilm formation, but demonstrated also potent anti-staphylococcal activity [69].

### 4.9. Isoquinoline Derivatives

Berberine chloride (Figure 8) was isolated from *Coptis chinensis* rhizome extract (chinese goldthread, Fam. Ranunculaceae) as one of the active compounds [80] responsible for the extract’s Sa-SrtA inhibition (IC_50_ = 16.7 μg/mL) [32]. Palmatine, the dimethoxy analog of berberine, and hydrastine, a related phthalide-isoquinoline derivative, were also evaluated as Sa-SrtA inhibitors. Berberine chloride registered an IC_50_ value of 23.4 μM and a MIC value of 269 μM on *S. aureus,* and over 1000 μM on *S. epidermidis*. Palmatine chloride presented an IC_50_ of 32.7 μM and a MIC value over 1000 μM on both *S. aureus* and *S. epidermidis* [80]. Berberine chloride is often used as a positive control in other Sa-SrtA inhibition assays, and depending on the specific methods used, the reported IC_50_ value were 85.9 μM [81], 99.8 μM [49], or 120 μM [82]. The Sa-SrtA inhibitory effect of palmatine was confirmed in another assay (IC_50_ = 52.8 μM) correlated with a low toxicity in the *Daphnia magna* test [28]. Coptisine chloride, the dioxolane analogue of berberine, inhibited the activity of Sa-SrtB (IC_50_ = 24.6 μM) without any visible antibacterial effects on *S. aureus* 29213 [83].

Three aaptamines alkaloids (Figure 8) isolated from the marine sponge *Aaptos aaptos* (Fam. Suberitidae) were identified as Sa-SrtA inhibitors. Isoaaptamine had the highest inhibitory effect (IC_50_ = 16.22 μM), probably due to the presence of the *N*-methyl group. Its isomer, aaptamine had an IC_50_ value of 102.9 μM, while its *N*-demethyl analog, demethylaaptamine, had an IC_50_ value of 85.9 μM. Isoaaptamine showed a moderate growth inhibition on *S. aureus* strain Newman with a MIC value of 219 μM [84].

### 4.10. Aryl β-Aminoethyl Ketones

**Figure 9:**
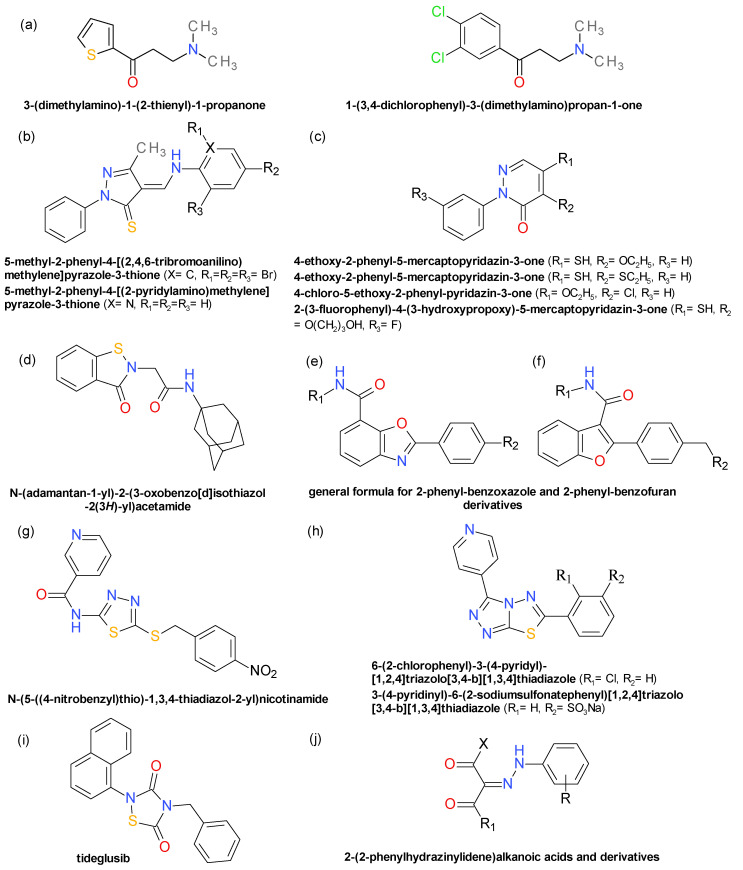
Representative sortase inhibitors from various chemotypes: (**a**) aryl β-aminoethyl ketones; (**b**) pyrazolethiones; (**c**) pyridazinones; (**d**) benzisothiazolinones; (**e**) 2-phenyl-benzoxazole; (**f**) 2-phenyl-benzofuran; (**g**) thiadiazoles; (**h**) triazolothiadiazoles; (**i**) 1,2,4-thiadiazolidine-3,5-dione; (**j**) 2-(2-phenylhydrazinylidene)alkanoic acids.

A series of SrtA inhibitors emerged after a screening of a 135,625 small molecules library using the fluorescence resonance energy transfer substrate method. A number of 6154 compounds displayed over 20% percentage inhibitions and were subjected to selection based on reactivity, genotoxic potential, and drug-like properties leading to 407 compounds. The compounds in this set were tested against papain, a eukaryotic protease with an active site thiol, in order to remove nonselective agents. Two aryl *β*-aminoethyl ketones, 3-(dimethylamino)-1-(2-thienyl)-1-propanone (AAEK1) and 1-(3,4-dichlorophenyl)-3-(dimethylamino)propan-1-one (AAEK2), were selected for further investigation (Figure 9). The compounds had IC_50_ values of 47 µM and 15 µM, respectively, for Sa-SrtA of *S. aureus*. AAEK1 and AAEK2 showed better inhibition of the SrtA homologue from *B. anthracis* with IC_50_ values of 4.8 µM, respectively 5.6 µM [85]. Mass spectrometry and X-ray crystallography studies of AAEK1 and SrtA interaction revealed that the inhibition mechanism is based on the elimination of the dimethylamino group and formation of the thienyl vinyl ketone that covalently binds to the enzyme Cys’s thiol [85].

### 4.11. Pyrazolethiones and Pyridazinones

A high-throughput screening assay on Sa-SrtAΔN59 identified several lead compounds as potent SrtA inhibitors. The research was extended on commercially available structurally similar compounds or newly synthesized ones belonging to the class of pyrazolethiones and the class of pyridazinones. The pyrazolethione derivatives presented IC_50_ in the range of 0.30 µM to 115 µM. The importance of the thione group was demonstrated by the decrease of the potency after its replacement with a ketone group and by the docking studies. The most active compounds (Figure 9) were 5-methyl-2-phenyl-4-[(2,4,6-tribromoanilino)methylene]pyrazole-3-thione (IC_50_ = 0.30 μM) and 5-methyl-2-phenyl-4-[(2-pyridylamino)methylene]pyrazole-3-thione (IC_50_ = 0.76 μM), both with a low effect on *S. aureus* growth at the tested concentration of 500 μM [86].

Some of the tested pyridazinone derivatives presented IC_50_ in the range of 0.20 µM to 219 µM, but for the majority of them the IC_50_ value is described as over the 50 μM threshold. The nature of the substituents on the pyridazinone ring influenced considerably the inhibitory activity. The best results were registered for 4-ethoxy-2-phenyl-5-mercaptopyridazin-3-one (IC_50_ = 0.20 μM), its 4-ethylsulfanyl analogue (IC_50_ = 1.4 μM), and 4-chloro-5-ethoxy-2-phenyl-pyridazin-3-one (IC_50_ = 1.0 μM) (Figure 9). At 500 μM 4-ethylsulfanyl-2-phenyl-5-mercaptopyridazin-3-one produced no effect on *S. aureus* growth, but the other 2 compounds almost totally inhibited the bacterial growth at the same concentration [86]. NMR spectroscopy, mass spectrometry, and docking studies revealed that the 5-mercaptopyridazin-3-one derivatives covalently modify Sa-SrtA by forming a disulfide bond with Cys184. A series of new pyridazinone derivatives were identified as potent Sa-SrtA inhibitor, 2-(3-fluorophenyl)-4-(3-hydroxypropoxy)-5-mercaptopyridazin-3-one emerging as the most potent inhibitor with an IC_50_ of 0.021 μM (Sa-SrtA) and of 0.45 μM (Ba-SrtA) [87].

### 4.12. Benzisothiazolinones

A high-throughput screening assay on Sa-SrtAΔN59 identified several lead compounds as potent SrtA inhibitors. *N*-(adamantan-1-yl)-2-(3-oxobenzo[d]isothiazol-2(3*H*)-yl)acetamide (Figure 9) was identified as a lead molecule that irreversibly inhibited SrtA (IC_50_ = 6.11 μM) by forming a disulphide bond with the Cys184 residue. Based on its structure, a series of related derivatives were synthesized and tested. The compounds presented IC_50_ values in the range of 3.39 µM to 7.06 µM and MIC values measured against *S. aureus* ranging from 20.02 µM to 163.91 µM. The majority of the derivatives had significant cytotoxic effects on mouse embryo fibroblast cell line NIH 3T3, limiting their therapeutically development [88].

### 4.13. Derivatives of 2-Phenyl-Benzoxazole and 2-Phenyl-Benzofuran

A series of 2-phenyl-benzo[d]oxazole-7-carboxamide derivatives were designed to mimic the shape and bonding properties of the characteristic SrtA’s motif LPXTG. The compounds presented IC_50_ values on Sa-SrtAΔN24 in the range of 19.8 µM to 184.2 µM. The nature of the substitution on the *para* position of the phenyl fragment has a great influence on the inhibitory activity, the best results being observed for a hydroxyl group esterified with various phenolic acids. The *N*-alkyl-benzoxazole-7-carboxamide scaffold functions as a structural analog of the L-leucyl-L-prolyl fragment of SrtA’s substrates and its importance was demonstrated by the low inhibitory effects of the compounds with no substituent in 7-position (IC_50_ > 200 µM) [89].

Based on the structure of the 2-phenyl-benzoxazole-7-carboxamide derivatives, a series of N-alkyl-2-phenyl-benzofuran-3-carboxamide derivatives (Figure 9) were synthesized and tested on Sa-SrtA registering IC_50_ values from 30.8 µM up to over 200 µM. Similar to the related benzoxazole derivatives, the best inhibitory effects were observed for the N-*i*-butyl-carboxamide derivatives and for those containing a dihydroxybenzoate moiety [90]. There are no data on the bacteria growth impact of these benzoxazole and benzofuran derivatives in order to evaluate their potential development as anti-virulence solutions.

### 4.14. Thiadiazoles Derivatives. Triazolothiadiazoles Derivatives

The compounds of this class share a common 1,3,4-thiadiazole central scaffold and an “L” geometry that is similar to the 2-phenyl-benzoxazole-7-carboxamide derivatives and their analogues 2-phenyl-benzofuran-3-carboxamide derivatives. These similarities are not necessary reflected in a common mechanism of SrtA inhibition.

High-throughput screening for Sa-SrtA inhibitors on a library of close to 28,500 compounds identified 5-[(7-nitro-2,1,3-benzoxadiazol-4-yl)sulfanyl]-1,3,4-thiadiazol-2-amine as a potent SrtA inhibitor (IC_50_ = 6.2 µM). The oxadiazole scaffold was removed because of the cytotoxicity risk associated with its presence and a series of derivatives of 5-amino-1,3,4-thiadiazole-2-thiol were synthesized and tested. The benzyl sulfide substituted derivatives demonstrated the best inhibitory effect, the *N*-(5-((4-nitrobenzyl)thio)-1,3,4-thiadiazol-2-yl)nicotinamide (IC_50_ = 3.8 µM) emerging as the optimal compound of this series (Figure 9). The experimental data suggest that this compound is reduced by the enzyme to a nicotinamide-thiadiazole thiol fragment that then forms a covalent disulphide bond at the Cys184 residue [91].

Based on the bis(indolyl)imidazole scaffold of topsentins derivatives, a molecular docking study on drug-like structures coupled with an experimental validation assay on Sa-SrtAΔN24 identified 6-(2-chlorophenyl)-3-(4-pyridyl)-[1,2,4]triazolo[3,4-b][1,3,4]thiadiazole (Figure 9) as a potent inhibitor (IC_50_ = 37.7 µM). The synthesis and testing of several analogues led to the optimized 3-(4-pyridinyl)-6-(2-sodiumsulfonatephenyl)[1,2,4]triazolo[3,4-b][1,3,4]thiadiazole (Figure 9), compound that reversibly inhibited SrtA with an IC_50_ of 9.3 µM. The compound registered a MIC value of over 40,000 µM on *S. aureus* in the microtiter broth dilution method. Administered doses of 40 mg/Kg in 12 h intervals for a period of 5 days in BALB/c mice infected with *S. aureus* Newman improved the 20-days survival rate at 53.3%, compared with 0% in the non-treated animals [91].

### 4.15. 1,2,4-Thiadiazolidine-3,5-Dione Derivatives

A screening for Sa-SrtA inhibitors on a collection of close to 2400 clinical drugs and candidates led to the identification of tideglusib (IC_50_ = 0.6 µM), an irreversible non-ATP-competitive glycogen synthase kinase 3β (GSK-3β) inhibitor [92]. Interestingly, the proposed GSK-3β inhibition mechanism, even if not unequivocally demonstrated, involves the enzyme’s Cys199 residue [93] and it could be similar in the SrtA case. Tideglusib minimally inhibited the growth of the *S. aureus* Newman strain. A series of thiadiazolidinedione derivatives were synthetized and tested to identify relevant structure activity relationships and to improve the anti-virulence profile. The replacement of the 2-naphtyl group with an ethyl, or benzyl, or phenethyl decreased the inhibitory activity on Sa-SrtA and considerably enhanced the antibacterial effects. The substitution of the naphtyl with a 3,5-dimethylisoxazole ring retained the SrtA inhibition, coupled with a better water solubility. The treatment with tideglusib in a dose of 40 mg/kg/day of BALB/c mice infected with *S. aureus* USA300 improved the 10-days survival rate to 40%, as to 10% in non-treated animals [92].

### 4.16. 2-(2-Phenylhydrazinylidene)Alkanoic Acids and Derivatives

A series of 1,3-dicarbonyl-2-phenylhydrazinylidene derivatives (Figure 9) were synthesized and evaluated on *S. aureus*’s SrtAΔN59-6His enzyme. The best inhibition was registered for the 3-oxo-2-(2-(3,4-dichlorophenyl)hydrazinylidene)butanoic acid showing an IC_50_ value of 50 µM. At 100 µM, the compound produced a 39% inhibition of the biofilm formation of *S. aureus* 25923, and a 71% reduction in the case of *S. aureus* 29213. The compounds had no important effect on *S. aureus* development, with MIC over 270 µM [94]. The compounds of this class are structurally similar to the pyrazolethione derivatives, indicating a potential common mechanism of SrtA inhibition.

### 4.17. Various Structures

**Figure 10:**
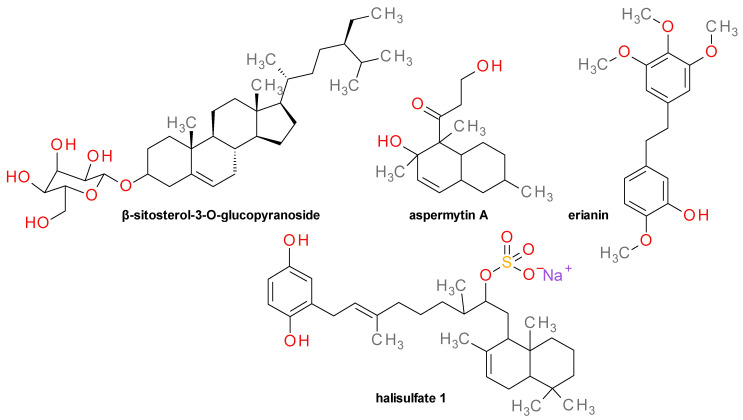
Sortase inhibitors with various structures.

A number of Srt inhibitors have unique chemical scaffolds and are difficult to enclose to a certain class. Here are presented some relevant compounds with diverse structures (Figure 10).

β-Sitosterol-3-*O*-glucopyranoside has been isolated from the bulbs of *Fritillaria verticillata* as Sa-SrtA inhibitor (IC_50_ = 31.7 µM), but with little activity against Sa-SrtB [25,57]. The compound demonstrated reduced antimicrobial activity against *S. aureus* with MIC values of 693 µM. Sitosterol was found to be inactive on SrtA and also on bacterial cell growth, indicating the importance of the glucoside moiety [25]. Aspermytin A, a polyketide isolated from the culture of the marine-derived fungus *Aspergillus sp.* F452 inhibited Sa-SrtA (IC_50_ = 146 µM) without a significant activity against *S*. *aureus* ATCC6538p [81]. Erianin, a natural dibenzyl compound, inhibited Sa-SrtA (IC_50_ = 65.7 µM) lacking any antibacterial effect on *S. aureus* (MIC ~ 1600 µM). The *S. aureus*-inoculated mice treated with 50 mg/Kg erianin administered three times a day for a period of 3 days had a 9-days survival rate of 30%, compared with 0% in the non-treated animals [95]. Halisulfate 1 was isolated from the sponge *Coscinoderma sp.* (Fam. Spongiidae) and demonstrated potent inhibitory effects on Sa-SrtA (IC_50_ = 36 µM), but also strong antibacterial effects on *S. aureus* [96].

## 5. Conclusions

A large diversity of Srt inhibitors is known. Generally, they are classified depending on their source of origin: natural or synthetic. The focus of this review was to identify and classify Srt inhibitors based on their structural features, significant for enzymatic inhibition. The IC_50_ values are presented as an indicator of each compound’s anti-virulence potential coupled, when available, with MIC values as a measure of the resistance risks. We classified the inhibitors based on their mechanistic relevant scaffolds: vinyl sulfones, 3-aryl acrylic acids and derivatives, flavonoids, naphtoquinones, anthraquinones, indoles, pyrrolomycins, isoquinoline derivatives, aryl β-aminoethyl ketones, pyrazolethiones, pyridazinones, benzisothiazolinones, 2-phenyl-benzoxazole and 2-phenyl-benzofuran derivatives, thiadiazoles, triazolothiadiazoles, 2-(2-phenylhydrazinylidene)alkanoic acids, and 1,2,4-thiadiazolidine-3,5-dione. We highlighted the structure–activity relationships of the inhibitors using IC_50_, when possible, for assessing the effect of each compound on a specific SrtA.

In a time of emerging bacterial resistance, sortases represent promising molecular targets for developing new antibiotics: a Srt inhibitor possesses a clear potential for being used as an alternative or complementary treatment of Gram-positive bacterial infections. Even if no candidates were promoted to clinical testing, this strategy could lead to the extension of the existing clinical antibacterial pipeline. We consider that an essential first step consists in having extensive knowledge on the already existing classes of inhibitors and understanding how certain structural features impact their efficacy as enzymatic inhibitors—efficacy expressed through IC_50_’s value. The purpose of this review was to offer such information, in a concise and accurate manner, as a useful tool for developing selective and effective Srt inhibitors.

## Data Availability

No new data were created or analyzed in this study. Data sharing is not applicable to this article.
